# The Kinetic Response of the Proteome in A549 Cells Exposed to ZnSO_4_ Stress

**DOI:** 10.1371/journal.pone.0133451

**Published:** 2015-07-21

**Authors:** Wen-jie Zhao, Qun Song, Zi-jin Zhang, Li Mao, Wei-juan Zheng, Xin Hu, Hong-zhen Lian

**Affiliations:** 1 State Key Laboratory of Analytical Chemistry for Life Science, Collaborative Innovation Center of Chemistry for Life Sciences, School of Chemistry & Chemical Engineering and Center of Materials Analysis, Nanjing University, Nanjing, Jiangsu, PR China; 2 MOE Key Laboratory of Modern Toxicology, School of Public Health, Nanjing Medical University, Nanjing, Jiangsu, PR China; 3 State Key Laboratory of Pharmaceutical Biotechnology, School of Life Science, Nanjing University, Nanjing, Jiangsu, PR China; Henan Agricultural Univerisity, CHINA

## Abstract

Zinc, an essential trace element, is involved in many important physiological processes. Cell responses to zinc stress show time-dependent effects besides concentration-dependence and tissue-specificity. Herein, we investigated the time-dependent differential expression of the proteome in A549 cells after administered with ZnSO_4_ for both 9 and 24 h using 2DE. 123 differentially expressed protein spots were detected, most of which were up-regulated by Zn^2+^ treatment. Interestingly, 49 proteins exhibited significant differential expression repeatedly during these two treatment periods, and moreover showed a conserved change with different ratios and four time-dependent expression patterns. Pattern 1 (up-regulated with rapid initial induction and subsequent repression) and pattern 4 (down-regulated with steady repression) were the predominant expression patterns. The abundances of the proteins in patterns 1 and 4 after 24 h of zinc treatment are always lower than that after 9 h, indicating that exogenous zinc reduced the expression of proteins in cells after 24 h or longer. Importantly, these findings could also reflect the central challenge in detecting zinc homeostasis proteins by 2DE or other high throughput analytical methods resulting from slight variation in protein expression after certain durations of exogenous zinc treatment and/or low inherent protein content in cells. These time-dependent proteome expression patterns were further validated by measuring dynamic changes in protein content in cells and in expression of two proteins using the Bradford method and western blotting, respectively. The time-dependent changes in total zinc and free Zn^2+^ ion contents in cells were measured using ICP-MS and confocal microscopy, respectively. The kinetic process of zinc homeostasis regulated by muffling was further revealed. In addition, we identified 50 differentially expressed proteins which are predominantly involved in metabolic process, cellular process or developmental process, and function as binding, catalytic activity or structural molecule activity. This study further elucidates our understanding of dynamic nature of the cellular response to zinc stress and the mechanism of zinc homeostasis.

## Introduction

Metal ions play fundamental roles in biology by serving as essential cofactors in diverse processes, such as respiration, growth, gene transcription, enzymatic reactions, cell proliferation, and immunity [1]. Cells in all organisms have mechanisms that maintain a constant cytosolic and organellar metal level to protect cells against metal toxicity and stress, whereas extracellular or dietary metal levels can fluctuate [2], [3]. Zinc, a widely distributed and rich essential trace element, participates in many important physiological functions, such as catalytic and structural activities [4–6]. Systems to balance zinc levels are controlled by metal regulatory transcription factors, zinc-binding proteins and zinc transporters, which maintain intracellular zinc homeostasis and are vital for normal cell function [7], [8]. The processes of maintaining zinc homeostasis primarily relies upon the control of zinc uptake, zinc efflux, and zinc binding within the cytosol or in organelles by proteins such as solute-carrier-39 A1 (SLC39A1, ZIP-1), SLC30A1 (ZnT-1), and metallothionein 1 (MT-1) [9], [10]. Metal responsive transcription factor 1 (MTF-1) is a zinc-responsive transcriptional activator that functions as a “toggle switch” for zinc homeostasis and induces the transcription of its target zinc-response genes [11]. However, excess or deficient zinc can cause oxidative stress and is toxic [2], [11]. Altered zinc homeostasis can lead to various diseases, including Alzheimer’s disease, prostate cancer, acquired immune deficiency syndrome (AIDS), diabetes, and alcoholic liver disease (ALD) [12–17].

Fortunately, all living organisms can cope with a variety of stressful situations by adapting their gene and/or protein expression [18]. The response of cells to zinc stress exhibits concentration-dependence, time-dependence, and tissue-specificity [19], [20]. Therefore, cells rely upon a network of zinc-responsive proteins to minimize intracellular zinc concentrations, while ensuring that sufficient zinc ions are present to facilitate zinc-dependent cellular processes and enzymatic reactions. The identification of ~3000 human zinc proteins was a major contribution to our understanding of the composition of the zinc-associated proteome and the functions of zinc-binding proteins [21]. Recently, many new findings have broadened our view of zinc homeostasis and toxicity, although major questions regarding multiple signaling pathways and the complex effects of intracellular zinc remain. The search for MTF-1 target genes, other than metallothionein genes, has been performed in human, mouse, and Drosophila systems [22]. A novel zinc transporter, ZIP10 (SLC39A10), involved in zinc uptake has been identified [23]. The iron-export ferroxidase activity of β-amyloid precursor protein was found to be inhibited by zinc in Alzheimer’s disease [24].

Examining changes in the expression of individual proteins or groups of proteins affected by metals stress is useful for gaining insight into the biomolecular mechanisms of zinc homeostasis or toxicity, and to identify potential candidate protein markers of metal exposure and response [25]. It is worth noting that proteomics analysis of the proteins expressed by a genome represents a powerful tool for both describing complete proteomes in organisms, and in comparing proteomes that are affected by different physiological conditions [26]. Such studies have yielded important large-scale datasets with utility for interpreting the roles of zinc in health and disease at both the molecular and global systems biology levels [21]. At the protein level, although newer technologies offering improved resolution and protein identification ability are regularly introduced, two-dimensional electrophoresis (2DE) remains the most accepted, widespread, and successfully implemented technique for the quantification, high-resolution separation, and characterization of these critical molecules [27]. Additionally, other “omics” (i.e., genomics, metabolomics, or lipidomics) tools or systematic analytical methods also provide high throughput strategies for investigating the biomolecular mechanisms of metal homeostasis or toxicity [28], [29]. For example, Barkla et al. carried out a quantitative proteomics analysis using two dimensional difference gel electrophoresis (2D-DIGE) to identify zinc-responsive proteins in *Arabidopsis thaliana* leaves after 4 days of 200 μM Zn^2+^ treatment [30]. Yamamoto and Ishihama used DNA microarray and S1 mapping assays to study the transcriptional response of *Escherichia coli* to extracellular zinc at various concentrations after treatment for 5 min [28]. Wu et al. detected metabolic differences between control and heavy metal-exposed (including zinc) clam samples after exposure for 48 h, utilizing a high resolution proton nuclear magnetic resonance (HR-^1^H NMR)-based metabolomics technique, and identified one sensitive pedigree (i.e. White clam) as a biomonitor for heavy metals [31].

The concentration-dependence and tissue-specificity of the response of cells to zinc stress has been extensively studied, by our own group and others, but the kinetic effects of zinc treatment on cells has received little attention [32], [33]. Moreover, most studies designed to investigate zinc homeostasis and toxicity have focused on the differential expression of a specific protein or proteome under a fixed stimulus period, such 24 or 48 h, which are the time-points that have been frequently used for 2DE analysis [28–31]. In such assays, some important differentially expressed proteins would be missed. Therefore, clarifying the kinetic effects of zinc treatment could yield important insights and aid our understanding of the molecular mechanisms of metal exposure, including zinc homeostasis and toxicity. Although some reports have described alterations of selective proteomes at different time points after metal treatment, the significant effects of metal stimulus period on the molecular response to metal stress has received very little attention to date. For example, Yıldırım et al. reported the differentially expressed proteins in mid-exponential phase cells subjected to 50 μM lead at four different exposure time points, but they did not further elucidate effects of exposure period on the molecular response [34].

Previously, we investigated time-dependent dynamic responses to Zn^2+^ treatment of key zinc homeostasis proteins, such as MT-1, MTF-1, ZnT-1, and ZIP-1, whose expressions in protein and mRNA levels exhibited maximum changes at 8–10 h during 0–24 h of ZnSO_4_ treatment [35]. We used A549 cells (a human lung adenocarcinoma cell line), which have been extensively used in the proteomic analysis of cells, as a model cell type. Herein, we profiled the global changes of proteome in A549 cells in response to extracellular zinc ions at different time points using a proteomics approach coupled with a more sensitive silver stain than previous Coomassie Brilliant Blue (CBB) stain. Notably, we concluded four time-dependent expression patterns of differentially expressed proteins in A549 cells. Additionally, we studied the time-dependent variation of proteins and zinc content in cells using Bradford, confocal imaging or inductively coupled plasma mass spectrometer (ICP-MS), aiming to further elucidate the processes and mechanisms involved in zinc homeostasis.

## Materials and Methods

### Chemicals and materials

Fetal bovine serum (KGY009), incomplete culture medium (KGM1640SF), and the Nuclei and Cytoplasm Protein Extraction Kit (KGP150) were supplied by KeyGen Biotech (Nanjing, China). The Bradford protein assay kit, Cell Counting Kit-8 (CCK-8 Kit), western blocking buffer, primary antibody dilution buffer, and secondary antibody dilution buffer were from Beyotime Institute of Biotechnology (Haimen, China). Trypsin was from Promega (Madison, USA). Nonlinear immobilized pH gradient (IPG) strips were purchased from GE (Piscataway, USA). Chemicals used for 2DE were purchased from Amresco (Solon, USA). ZnSO_4_∙7H_2_O was purchased from Aladdin (Shanghai, China). All water used in experiments was Millipore Milli-Q filtered at a resistivity greater than or equal to 18.25 MΩ·cm. Culture dishes, and polyvinylidene difluoride (PVDF) membranes were from Millipore (Bedford, USA). Primary antibodies were purchased from the following vendors: anti-actin antibody (AA128, Beyotime); Hsp90α (D1A7) rabbit mAb (CST#8165) and hnRNPA1 (D21H11) rabbit mAb (CST#8443) (both Cell Signaling Technology). Secondary antibodies, including goat anti-rabbit lgG-HRP (sc-2004) and goat anti-mouse lgG-HRP (sc-2005), were purchased from Santa Cruz Biotechnology.

### Cell culture

A549 cells (human lung adenocarcinoma cell line) were purchased from KeyGen Biotech (Nanjing, China) and were maintained in incomplete culture medium supplemented with 10% fetal bovine serum, 80 units/mL penicillin, and 0.08 mg/mL streptomycin. Cells were routinely incubated at 37°C in a 5% humidified CO_2_-enriched atmosphere. For 2DE, western blot analysis, and determinations of proteins and zinc content, cells were grown in 100 mm diameter culture dishes. Cells were plated in 6-well plates at a density of 1×10^5^ cells/well for confocal imaging, and in 96-well plates at a density of 5×10^3^ cells/well for cell viability analyses. When cells reached ~80% confluence, they were harvested.

### Exogenous Zn^2+^ stress

A stock solution was prepared with ZnSO_4_∙7H_2_O. The salt was dissolved in Milli-Q water and sterile-filtered using a 0.22 μm filter. The concentration of zinc was determined to be 85.7 mM using a Perkin-Elmer OPTIMA 5300DV inductively coupled plasma-optical emission (ICP-OES) (MA, USA). A549 cells were treated for different times or concentrations of ZnSO_4_ dissolved in culture medium after cells reached ~30% confluence. Then, cells were harvested when cells reached ~80% confluence, and were washed twice with 3 mL pre-warmed (37°C) phosphate-buffered saline (PBS, pH 7.4) to remove any metal contamination derived from the culture medium. Following treatment, cultures were renewed with fresh culture medium containing 25, 50, 75, 100, 150, 200, 300, or 500 μM ZnSO_4_ for 9 or 24 h for cell viability analysis. A549 cells were treated with a non-physiological concentration of ZnSO_4_ (100 μM) dissolved in culture medium for 9 or 24 h for the 2DE experiment. Cells were subsequently incubated with 100 μM ZnSO_4_ for 6, 8, 10, 12, 24, or 48 h for the determination of protein and zinc content in the nucleus, cytoplasm, and whole cells. To observe changes in the abundance of differentially expressed proteins and the distribution of Zn^2+^, A549 cells were treated with 100 μM ZnSO_4_ for 6, 9, 12, or 24 h. Control groups were not treated with ZnSO_4_.

### Cell viability assays

CCK-8 was used to test the viability of A549 cells after different time-courses of treatment or exposure to various doses of ZnSO_4_. Approximately 5000 cells were seeded in each well. Cells were treated with various concentrations of ZnSO_4_ and incubated for 9 or 24 h. The medium was renewed with fresh culture medium containing identical concentrations of ZnSO_4_ before assay using the kit, for which 10 μL CCK-8 solution was added to each well, and the plate was incubated for 30 min to 1 h at 37°C. Absorbance was measured at 450 nm using a Bio-Rad 680 enzyme micro-plate reader after 45 min to obtain stable data. Cell viability assay was calculated by comparing absorbance value of cells treated and untreated with ZnSO_4_, and the cell viability of cells untreated was as 100%.

### Preparation of protein samples

Cells were harvested using ice-cold PBS and collected in 1.5 mL eppendorf tubes. Cell numbers were recorded using a hemocytometer. The number of cells was adjusted to ~1×10^7^ cells per mL in PBS. Whole proteins in cells were prepared by suspending the cell pellets with 200 μL lysis buffer [7 M urea, 2 M thiourea, 4% w/v 3-[(3-cholamidopropyl)dimethyl-ammonio]-1-propanesulfonate (CHAPS), 1% dithiothreitol (DTT), 2% v/v IPG (immobilized pH gradient) buffer nonlinear pH 3–10, 0.01% v/v phenylmethylsulfonyl fluoride (PMSF)], and were then vortexed vigorously at 4°C for 3 min. Lysates were sonicated for 1 min at a 10% amplitude at interval settings of 2 s on and 3 s off with a Sonicator S-4000 (Misonix, Shanghai, China). The heat generated by sonication was below 1000 J. The supernatants were clarified and recovered after centrifugation at 15,000×*g* for 30 min at 4°C. The fractionation of cells into nuclei and cytoplasm was performed using a Nuclei and Cytoplasm Protein Extraction Kit. The fractionation extraction steps were carried out according to the product specification. Protein samples were flash-frozen and stored at –80°C. The concentrations of the protein extracts were determined using the Bradford method [36].

### 2DE and image analyses

Protein separation was carried out using a GE Healthcare IPGphor IEF (isoelectric focusing) and an Ettan Dalt six electrophoresis system. Isoelectric focusing was performed using 24 cm precast nonlinear IPG strips (pH 3–10). Then, 200 μg whole cell proteins prepared as above were mixed with 450 μL rehydration buffer [7 M urea, 2 M thiourea, 4% w/v CHAPS, 1% DTT, 0.5% v/v IPG buffer nonlinear pH 3–10, and 0.002% bromophenol blue (BPB)] and loaded onto IPG strips by in-gel rehydration at room temperature overnight. IEF was performed using a step-wise voltage increase procedure at 20°C with the following parameters: 50 V for 12 h in Step (Stp) mode, 500 V for 1 h in Stp mode, 1000 V for 1 h in Gradient (Grd) mode, 10,000 V for 1 h in Grd mode, and 10,000 V for 100,000 vh in Stp mode, followed by 500 V Stp in holding mode.

After IEF, the IPG strips were subjected to a two-step equilibration [6 M urea, 30% glycerol, 2% sodium dodecyl sulfate (SDS), 0.002% BPB, 50 mM Tris-HCl, pH 8.8] with 1% DTT (w/v) for the first step and 2.5% iodoacetamide (w/v) for the second step. Separation in the second dimension was performed using 1 mm thick 12% polyacrylamide gels in Tris-glycine buffer (25 mM Tris-HCl, 192 mM glycine, 0.1% SDS, pH 8.3). Electrophoresis was carried out at 100 V for 45 min, and then at 200 V for ~4 h at 15°C until the BPB marker reached the bottom of the gel.

All samples including controls were analyzed in triplicate, and 9 gel pieces were visualized by silver nitrate staining according to protocol of Shevchenko et al. [37]. The limit of detection (LOD) of silver staining was as low as 1 ng per spot. Gels were then scanned using an ImageScanner (GE Healthcare, Piscataway, USA). Spot detection and quantification were carried out using PDQuest 8.0 analysis software (Bio-Rad, Hercules, USA). The numbers of protein spots were determined. The ratios of protein abundance were obtained by comparing the mean abundance in triplicate gels of corresponding differentially expressed proteins after treatment for 9 or 24 h with their controls using gel analysis software. Spots with at least two-fold differential expression (ratio values were higher than 2 and lower than 0.5 for up- and down-regulated proteins, respectively) between the ZnSO_4_ and control groups, and a *p*-value resulting from ANOVA analysis of less than 0.05 were considered to be significantly changed, and were subsequently subjected to differential expression analysis and protein identification.

### Protein identification

Protein spots of interest were manually excised from gels and digested with trypsin. Excised gel pieces were washed twice with Milli-Q water. Gel particles were destained with 15 mM potassium ferricyanide [K_3_Fe(CN)_6_] and 50 mM sodium thiosulfate (NaS_2_O_3_) at room temperature for 5 min, and then were covered by 50% acetonitrile (ACN). After removing the 50% ACN, 100% ACN was added to the gel particles for 5 min. Gel particles were incubated with 2–4 μL 20 μg/mL trypsin in 25 mM ammonium hydrogen carbonate for 30 min. In-gel digestion with trypsin was performed at 37°C for 16 h. Supernatants from the trypsin-digested mixtures were collected in separate tubes, and peptides were extracted once using 50 μL extraction buffer [67% ACN and 5% trifluoroacetic acid (TFA)]. All supernatants derived from the peptide extracts were mixed and then were completely dried.

Extracted peptide samples were analyzed on a 5800 Plus MALDI TOF/TOF (Matrix-assisted laser desorption ionization time-of-flight tandem mass spectrometer) Analyzer (Applied Biosystemss, Foster City, USA). Each sample was re-suspended in 5 μL 0.1% TFA, then was mixed at a 1:1 ratio with a saturated solution of α-cyano-4-hydroxycinnamic acid in 50% ACN and 0.1% TFA, and then was spotted onto MALDI targets. MS/MS ions were obtained in positive reflector mode using a CalMix5 standard (ABI 5800 Calibration Mixture) to calibrate the instrument. Proteins were successfully identified based on a 95% or greater confidence interval of their scores using the MASCOT V2.3 search engine (Matrix Science Ltd., London, UK) to query the human protein NCBI database using the following search criteria: 100 ppm peptide mass tolerance, 0.5 Da fragment mass tolerance for MS/MS, maximum of 1 missed cleavage, a fixed carbamidomethyl (C) modification, and acetyl (protein N-term), deamidated (NQ), dioxidation (W), and oxidation (M) variable modifications.

### Western blot analysis

Thirty micrograms of proteins in whole cell extracts were fractionated on 12% acrylamide gels by sodium dodecyl sulfate-polyacrylamide gel electrophoresis (SDS-PAGE) according to Laemmli’s method, and proteins were electrotransferred onto PVDF membranes using a Mini P-4 electrotransfer apparatus (Cavoy, Beijing, China) [38]. PVDF membranes were activated by soaking in methanol for 1 min prior to blotting. The membranes were then equilibrated for 10 min in blotting buffer [48 mM Tris-base, 39 mM glycine, 20% (v/v) methanol, and 0.0375 (w/v) SDS]. A “blotting sandwich” was made according to the manufacturer’s instructions. Blotting was carried out for 1 h on ice at a constant voltage of 100 V. After transfer, the membrane was blocked in western blocking buffer for 1–2 h at room temperature. After four washing steps with PBST (PBS with Tween-20) for 4 min, the membrane was incubated with primary antibody overnight at 4°C. The primary antibodies were diluted in primary antibody dilution buffer as ratio of 1:1000 according to manufacturer’s specifications. Then, the membrane was washed four times with PBST for 4 min and incubated for 1 h at room temperature in the presence of the appropriate horseradish peroxidase-conjugated secondary antibody. After several washes, the membrane was incubated with Pierce ECL Western Blotting Substrate (Thermo Scientific, Rockford, USA) and immune complexes were detected using the enhanced chemiluminescence assay (CLINX, Shanghai, China). After allowing adequate time for development, the reaction was stopped by rinsing with PBST. The low-background membrane was incubated with different antibodies and developed repeatedly. Scanning densitometry and quantitative analysis of immunoblot data were performed using dedicated Gel Image Analysis software (CLINX, Shanghai, China). β-actin was used as an internal control. Data were normalized and mean value ± standard error of the mean (SEM) was calculated from at least three independent samples.

### Determination of zinc concentration

The collection, lysis, and cell fractionation steps were described above in the “Preparation of protein samples” section. Then, cell lysates were digested for 7 h using HNO_3_ and H_2_O_2_ (2:5, v/v). The concentrations of zinc in whole cell, cytoplasm, and nuclear fractions were determined using a Perkin-Elmer Sciex Elan 9000 ICP-MS (Überlingen, Germany).

### Confocal imaging of intracellular Zn^2+^ with NBD-TPEA

N-(2-(bis((pyridin-2-yl)methyl)amino)ethyl)-7-nitro-N-((pyridin-2-yl)methyl)benzo[c][1,2,5]oxadiazole-4-amine (NBD-TPEA) is a visible and excitable cytoplasm-specific fluorescent probe that displays distinct and selective Zn^2+^-amplified fluorescence and is robustly excited and emitted at 469 and 550 nm, respectively [39]. The probe stably combines with free Zn^2+^ ions and forms 1:1 complexes, for which the emission intensity shows no significant changes at pH 7.1–10.1. The changes in intracellular Zn^2+^ were monitored with NBD-TPEA. A549 cells (1×10^5^ cells) were incubated in glass bottom cell culture dishes (Zhongjingkeyi Technology Co., Ltd., Beijing, China). ZnSO_4_ was added to the cultures at a concentration of 100 μM, and cells were incubated for an additional 6, 9, 12, or 24 h. Cells were then incubated with NBD-TPEA in the dark at room temperature for 30 min. After removing the excess NBD-TPEA, confocal imaging was performed on a Leica TCS-SP5 two-photon laser-scanning confocal microscope (Wetzlar, Germany). Pixels, scanning speed, and scan mode were set as 1024×1024, 200 Hz, and xyz mode, respectively. Images were captured at 488 nm excitation and 550 nm emission wavelengths. Quantification of Zn-NBD-TPEA fluorescence was performed using Image-ProPlus 6.0 software.

### Statistical analyses

All measurements were repeated at least three times and data were expressed as mean ± SEM. Statistical significance for comparison of two groups was assessed, unless otherwise specified, using one-way ANOVA with the Tukey–Kramer multiple comparison post-hoc test. Differences that were considered to be statistically significant are indicated as follows: *, *p*<0.05; **, *p*<0.01; and ***, *p*<0.001 vs. untreated controls.

## Results

### Cell viability

**Fig 1:**
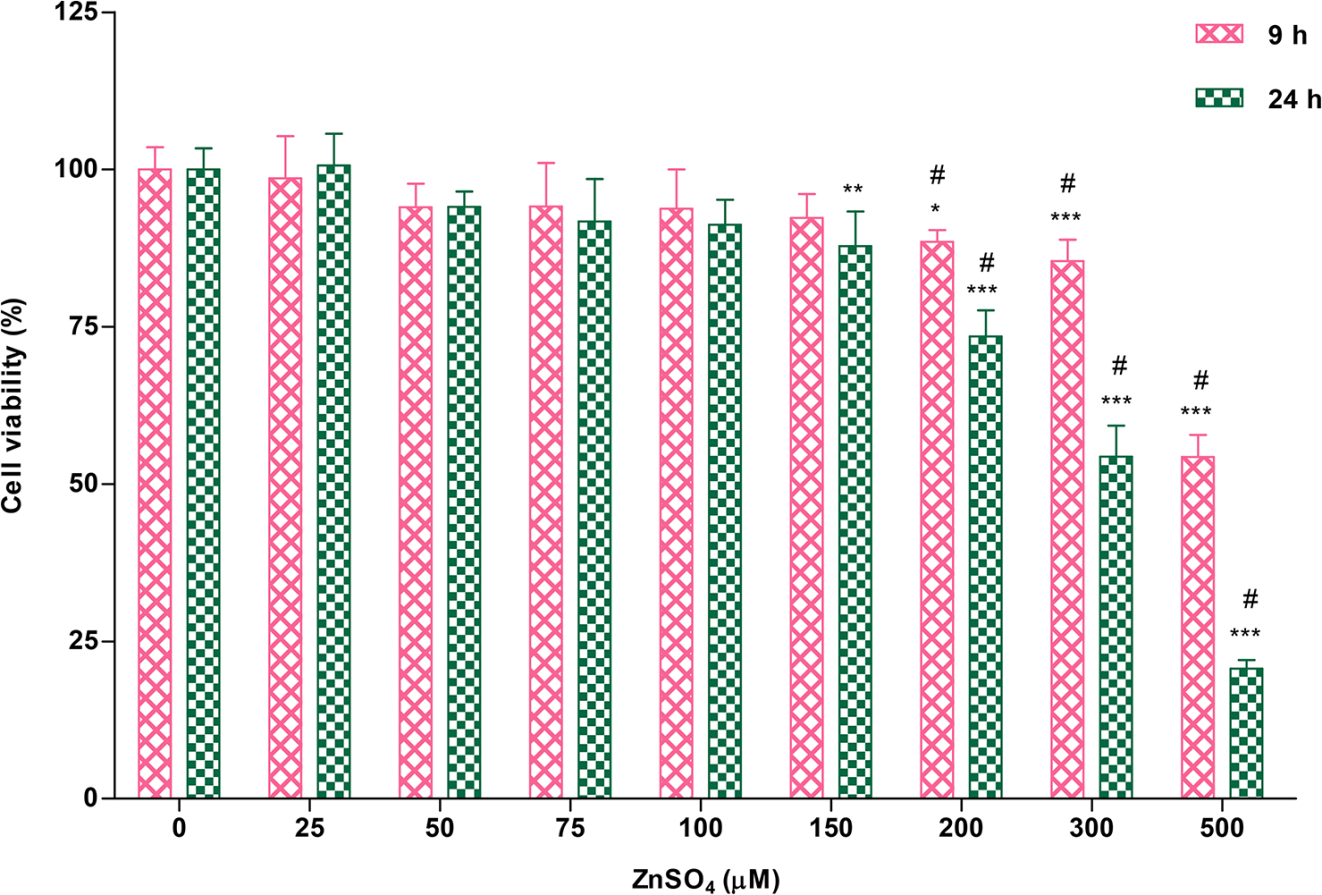
Viability of A549 cells after treatment with ZnSO_4_ for 9 or 24 h. The viability of A549 cells was assessed using CKK-8 after treatment with various doses of ZnSO_4_ for 9 or 24 h. Cell viability showed kinetic changes and was significantly decrease after treatment with 200 μM ZnSO_4_ for 9 h and with 150 μM ZnSO_4_ for 24 h. #, *p*<0.05, 9 h vs. 24 h by two-way ANOVA analysis.

The viability of A549 cells after treatment with various concentrations of ZnSO_4_ for 9 or 24 h was assayed (Fig 1). Elevated ZnSO_4_ concentrations reduced cell viability, although the viability of A549 cells remained about 50% and 20% after treatment with 500 μM ZnSO_4_ for 9 and 24 h, respectively. This A549 cell line presented more tolerance to exogenous zinc treatment, especially to above 200 μM ZnSO_4_, than the cell line from another vendor in our previous work [35]. Moreover, the viability of A549 cells showed a striking difference after treatment with ZnSO_4_ for 9 and 24 h at the concentration over 150 μM (#, *p*<0.05). Our statistical analyses indicated that the viability of cells significantly declined after 24 h of 150 μM Zn^2+^ treatment (**, *p*<0.01), whereas a significant difference in cell viability could be observed at an elevated concentration of ZnSO_4_ (i.e., 200 μM) after treatment for only 9 h (*, *p*<0.05). The viability of A549 cells declined by less than 10%, and was not significantly different between cells treated with 100 μM ZnSO_4_ for 9 or 24 h, indicating that 100 μM ZnSO_4_ represents a sub-cytotoxic metal concentration. Therefore, 100 μM ZnSO_4_ administered for appropriate periods of time was used for further analysis.

### Proteome expression patterns in response to Zn^2+^

**Fig 2:**
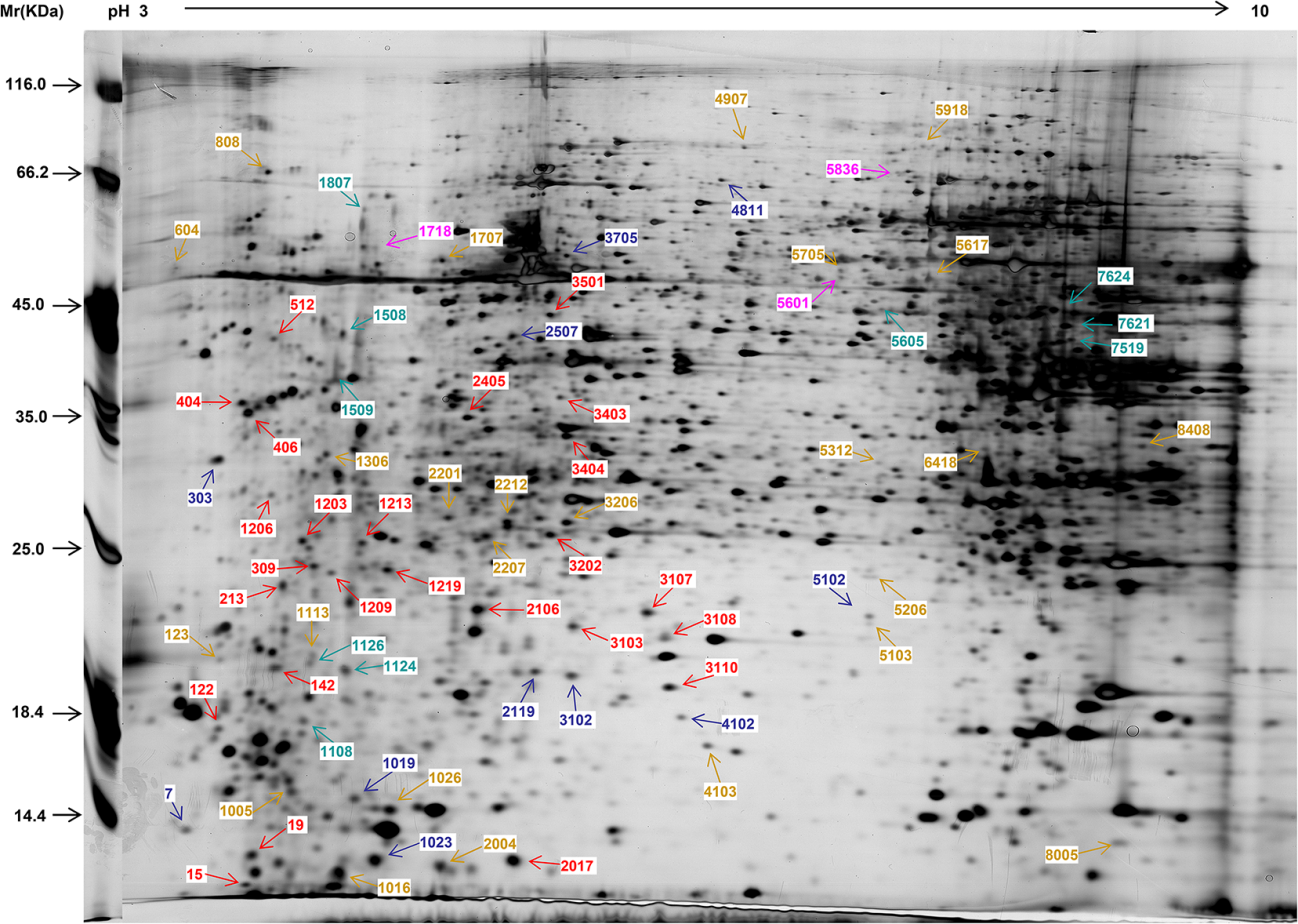
Representative 2DE gels of soluble proteins in A549 cells stained with AgNO_3_. A549 cells were incubated with 100 μM ZnSO_4_ for 9 or 24 h. Whole cell proteins were extracted as described in “Preparation of protein samples” in the Materials and Methods section. Controls were not treated with ZnSO_4_. Each protein sample (200 μg) was separated by IEF in a 24 cm IPG gel strip containing a broad nonlinear pH gradient of 3–10, followed by SDS–PAGE on a vertical 12% gel. Differentially expressed proteins that changed in response to 100 μM ZnSO_4_ for 9 or 24 h are illustrated with different colors. These proteins in A549 cells showed a two-fold or greater difference in abundance versus controls (*p*<0.05). Among 74 unique differentially expressed proteins spots, a total of 49 protein spots showed significantly differential responses to Zn^2+^ treatment at both 9 and 24 h, while other 25 significantly differentially expressed proteins spots were only observed after 9 or 24 h of Zn^2+^ treatment. Among the 49 protein spots, the up-regulated protein spots showing a larger or less change after 9 h of treatment compared to 24 h are marked by red and pink, respectively; the down-regulated protein spots showing a larger or less change after 24 h of treatment compared to 9 h are marked by blue and green, respectively. Other 25 significantly differentially expressed proteins spots after either 9 or 24 h are marked by brown.

To investigate the kinetic response of the proteome in A549 cells exposed to zinc stress, cells were exposed to 100 μM ZnSO_4_ for 9 or 24 h, and whole cell proteins were subjected to highly sensitive silver nitrate staining on 2DE gels in triplicate. A representative 2DE gel after Zn^2+^ treatment is shown in Fig 2. Comparisons of the three groups of gels (controls and Zn^2+^ treatment for 9 or 24 h) were performed. 17690 spots were detected on these gels and 2047 matches were mapped to a reference gel. Compared to controls, 68 and 55 protein spots (total of 123 protein spots) showed significantly differential expression (*p*<0.05, fold change>2) after treatment with 100 μM ZnSO_4_ for 9 or 24 h, respectively. All the 123 differentially expressed protein spots were divided in to two groups as summarized in S1 Fig and S1 Table, respectively. 49 protein spots showed significantly different responses to Zn^2+^ exposure at both 9 and 24 h (S1 Fig), and therefore, these proteins were used as our main study criteria. In addition, other 25 protein spots showed significantly different responses to Zn^2+^ exposure at both 9 or 24 h (S1 Table). Actually, there were a total of 74 unique different proteins spots in two groups of samples (9 or 24 h vs. control), which are indicated in Fig 2 (including the match numbers).

Among the 49 differentially expressed protein spots that existed in the two groups of samples after treatment for 9 and 24 h, the up- and down-regulated protein spots accounted for 73.5% (36 proteins) and 26.5% (13 proteins), respectively. Additionally, 25 protein spots (69.4%, marked by red in the representative gel) of the 36 up-regulated protein spots showed a larger change after 9 h of treatment compared to 24 h, which indicates that a longer period of stimulation would mainly decrease the expression of up-regulated proteins. By contrast, 10 (76.9%, marked by blue in the representative gel) of the 13 down-regulated protein spots showed a larger change after 24 h of treatment compared to 9 h, which also indicates that a longer stimulation would mostly further repress the expression of down-regulated proteins. Therefore, a total of 35 (71.4%) of the 49 protein spots exhibited lower expression after 24 h of treatment compared to 9 h, and longer stimulation mainly reduced the expression of differentially expressed proteins. These similar phenomena were also found among the other 25 significantly differentially expressed proteins after either 9 h or 24 h of Zn^2+^ treatment (see S1 Table, marked by brown in the representative gel). The rest 11 (30.6%, marked by pink in the representative gel) of the 36 up-regulated protein spots and 3 (23.1%, marked by green in the representative gel) of the 13 down-regulated protein spots varied irregularly in abundance after different treatment periods and accounted for small percentages.

**Fig 3:**
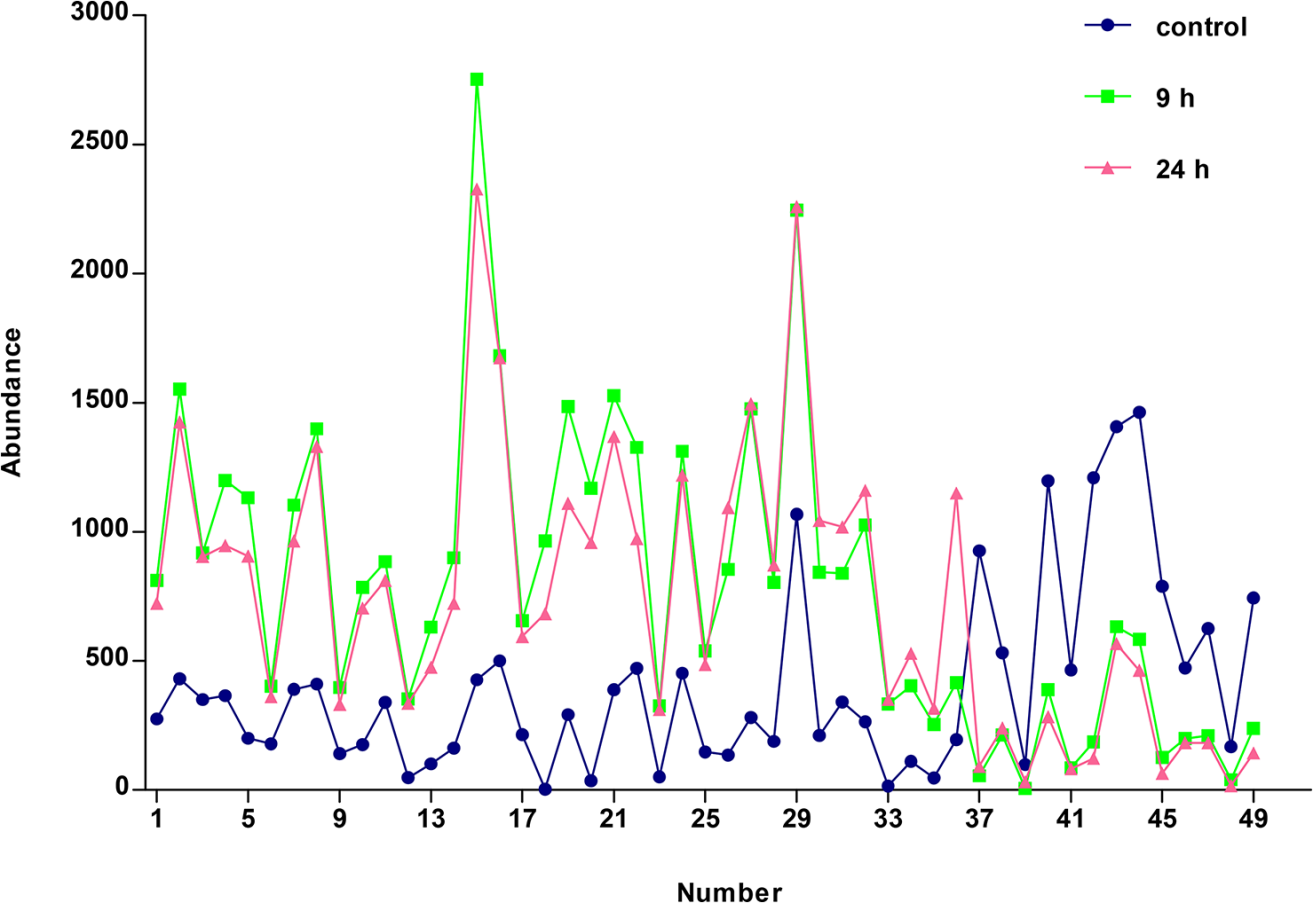
Abundances of differentially expressed proteins after Zn^2+^ treatment for both 9 and 24 h.

**Fig 4:**
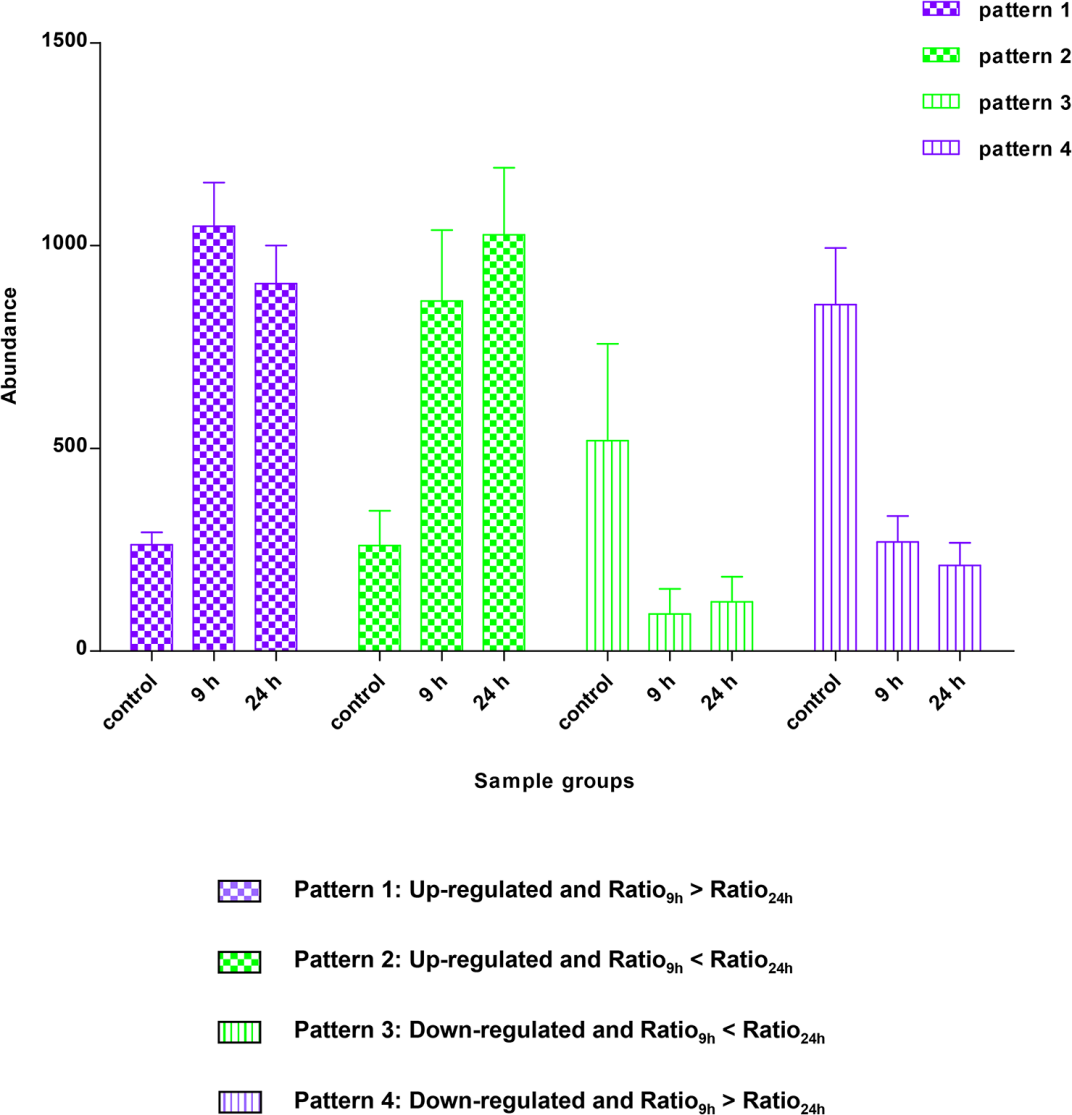
Expression patterns of differentially expressed proteins after Zn^2+^ treatment for both 9 and 24 h.

Mean abundance of each spot among the 49 protein spots after treatment with ZnSO_4_ for 9 or 24 h in triplicate samples is presented in Fig 3. Numbers 1–25 represent proteins which were up-regulated after Zn^2+^ treatment. Their abundance ratios compared with controls at 9 h were higher than those at 24 h (Ratio_9h_ > Ratio_24h_). Numbers 26–36 represent proteins which were up-regulated after Zn^2+^ treatment. Their abundance ratios compared with controls at 9 h were lower than those at 24 h (Ratio_9h_ < Ratio_24h_). Numbers 37–39 represent proteins which were down-regulated after Zn^2+^ treatment. Their abundance ratios compared with controls at 9 h were lower than those at 24 h (Ratio_9h_ < Ratio_24h_). Numbers 40–49 represent proteins which were up-regulated after Zn^2+^ treatment. Their abundance ratios compared with controls at 9 h were higher than those at 24 h (Ratio_9h_ > Ratio_24h_). The differential expression patterns of the 49 protein spots were classified into four models accordingly (i.e. patterns 1, 2, 3 and 4). The meanings of these four patterns were illustrated by corresponding histograms that are presented in Fig 4. The height of each column in histograms was mean abundance of all differentially expressed protein spots in corresponding groups.

### Identification and functional classification of proteins differentially expressed in response to Zn^2+^

**Fig 5:**
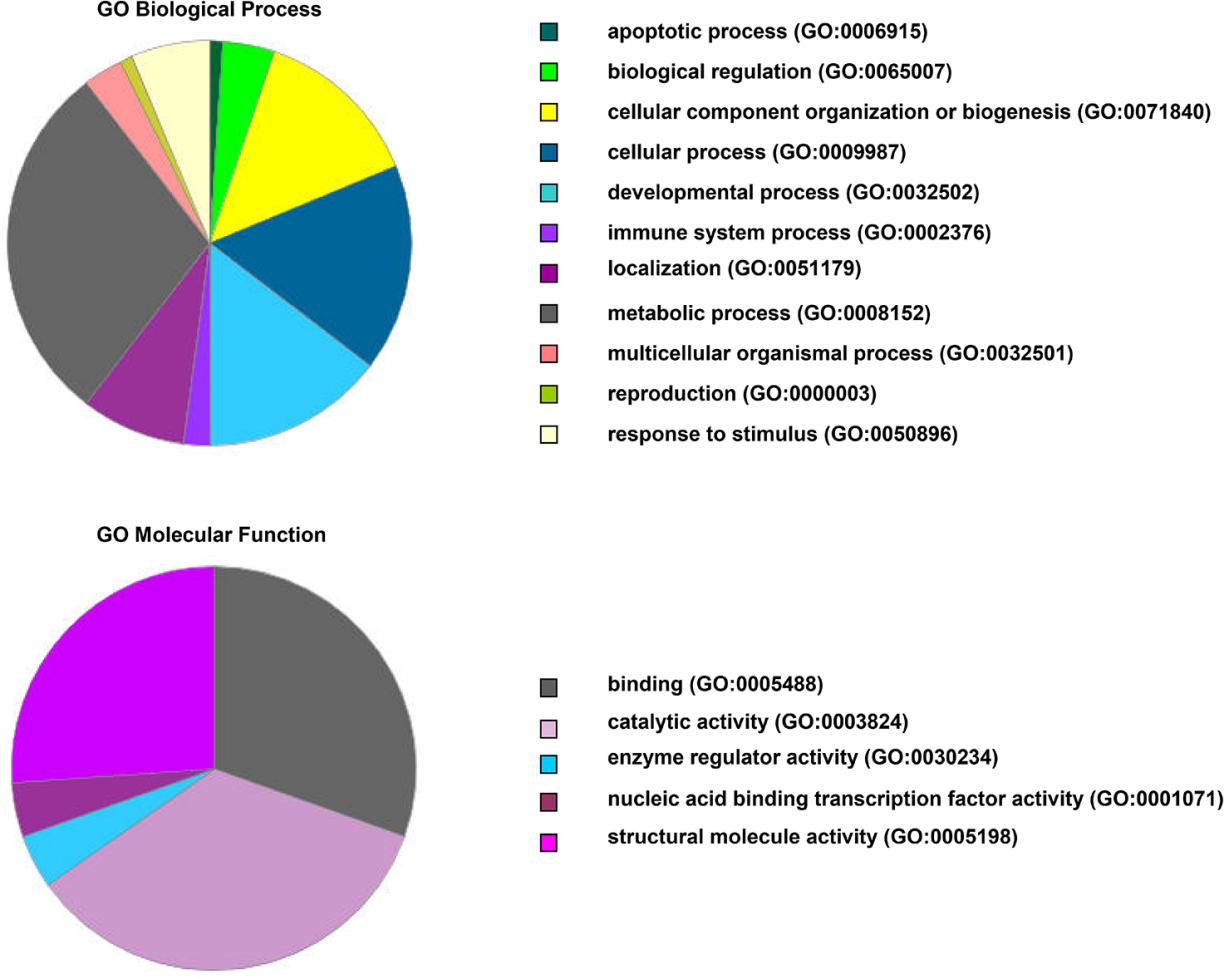
PANTHER classification of differentially expressed proteins identified by MS/MS. Biological process (upper) and molecular function (lower) classifications are shown.

MALDI TOF/TOF was used to identify zinc-responsive proteins that exhibit significantly differential expression patterns compared with controls in response to zinc treatment. Among 123 differentially expressed protein spots, 50 were successfully identified by MS/MS. These proteins are described in supplemental S1 File. Most of these proteins have been previously implicated in various intracellular physiological activities [40–42]. The differentially expressed proteins that we identified were categorized according to the PANTHER Classification System (http://pantherdb.org/). Among these proteins, 44 had a reliable “hit” within the system. These proteins were classified according to biological process (Fig 5) and were predominantly involved in the metabolic process (GO:0008152), cellular process (GO:0009987), developmental process (GO:0032502), and cellular component organization or biogenesis (GO:0071840) categories. Other biological processes accounted for a small percentage of the proteins identified, which included the localization (GO:0051179), response to stimulus (GO:0050896), biological regulation (GO:0065007), multicellular organismal process (GO:0032501), immune system process (GO:0002376), apoptotic process (GO:0006915), and reproduction (GO:0000003) categories. Moreover, the protein hits could also be classified according to molecular function (Fig 5). Catalytic activity (GO:0003824), binding (GO:0005488), and structural molecule activity (GO:0005488) were the most common molecular functions of the differentially expressed proteins. Enzyme regulator activity (GO:0030234) and nucleic acid binding transcription factor activity (GO:0001071) accounted for a smaller number of the molecular functions of these proteins.

### HSP90α and hnRNPA1 expression patterns

**Fig 6:**
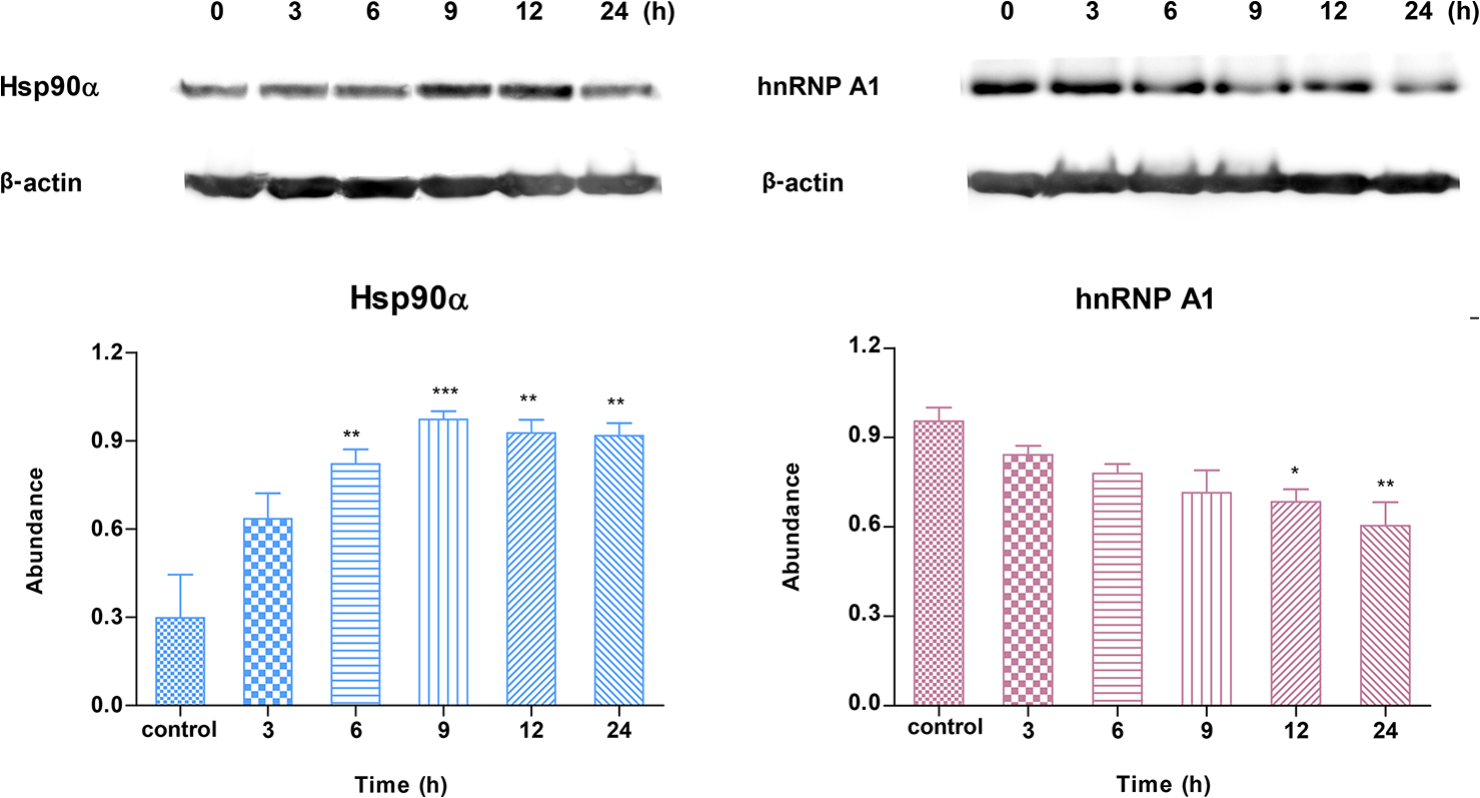
Expression patterns of Hsp90α and hnRNPA1 at the protein level. The abundances of proteins changed in time-dependent manner. Western blot (upper) and protein band density (lower) analysis of Hsp90α and hnRNPA1 protein levels in A549 cells after different periods of stimulation with exogenous 100 μM ZnSO_4_ were analyzed. The expression patterns of Hsp90α and hnRNPA1 at the protein level corresponded to prevailing patterns 1 and 4, respectively. Data were normalized to maximum values.

To confirm expression patterns of zinc-responsive proteins, the kinetic responses of heat shock protein 90 alpha (Hsp90α) and heterogenous nuclear ribonucleoprotein A1 (hnRNPA1) were observed when A549 cells were incubated with ZnSO_4_ for 3, 6, 9, 12, or 24 h. The relative abundances of proteins treated with Zn^2+^ compared to an internal reference protein were analyzed and normalized. These differential expression patterns are shown in Fig 6. Exposure to Zn^2+^ produced a peak value of Hsp90α after 9 h, which decreased after 12 h of treatment. Statistical analysis revealed significant differences in abundance of Hsp90α after treatment for 6, 9, 12, and 24 h. Hsp90α showed a robust kinetic response to Zn^2+^ treatment in a time-dependent manner. A different time-course of hnRNPA1 protein expression after Zn^2+^ treatment was also observed (Fig 6). The expression of hnRNPA1 could be suppressed by ZnSO_4_ treatment, and the expression gradually decreased.

### Differential protein concentrations

**Fig 7:**
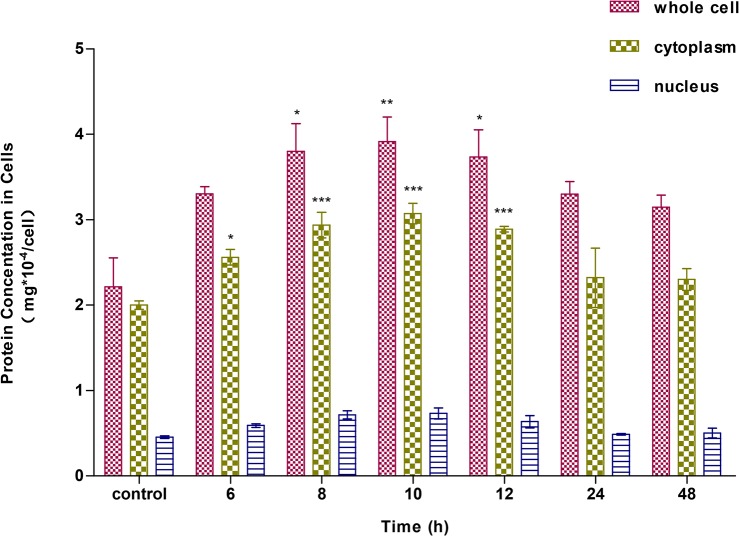
Differential concentrations of proteins in A549 cells. A549 cells were incubated with 100 μM ZnSO_4_ for 6, 8, 10, 12, 24, or 48 h. Cells were fractionated into cytoplasmic and nuclear fractions. Concentrations of proteins in whole cell, cytoplasm, and nucleus were determined, and kinetic changes were observed.

The concentrations of proteins in whole cells, cytoplasm, and nuclei of A549 cells treated with ZnSO_4_ for 6, 8, 10, 12, 24, or 48 h were determined by the Bradford method (Fig 7). The protein concentrations changed in a time-dependent manner. The total expression of proteins in whole cells, cytoplasm, and nuclei increased gradually after Zn^2+^ treatment, and reached a peak between 8 to 10 h, then decreased slowly. The protein concentrations in whole cells and cytoplasm between 24 and 48 h of treatment were not statistically different, suggesting that a plateau was reached after 24 h. There was no significant increase in protein concentration in nuclei, irrespective of the length of treatment, probably because the nuclear proteins were relatively less abundant and not easily affected by exogenous stimuli.

### Intracellular zinc accumulation

**Fig 8:**
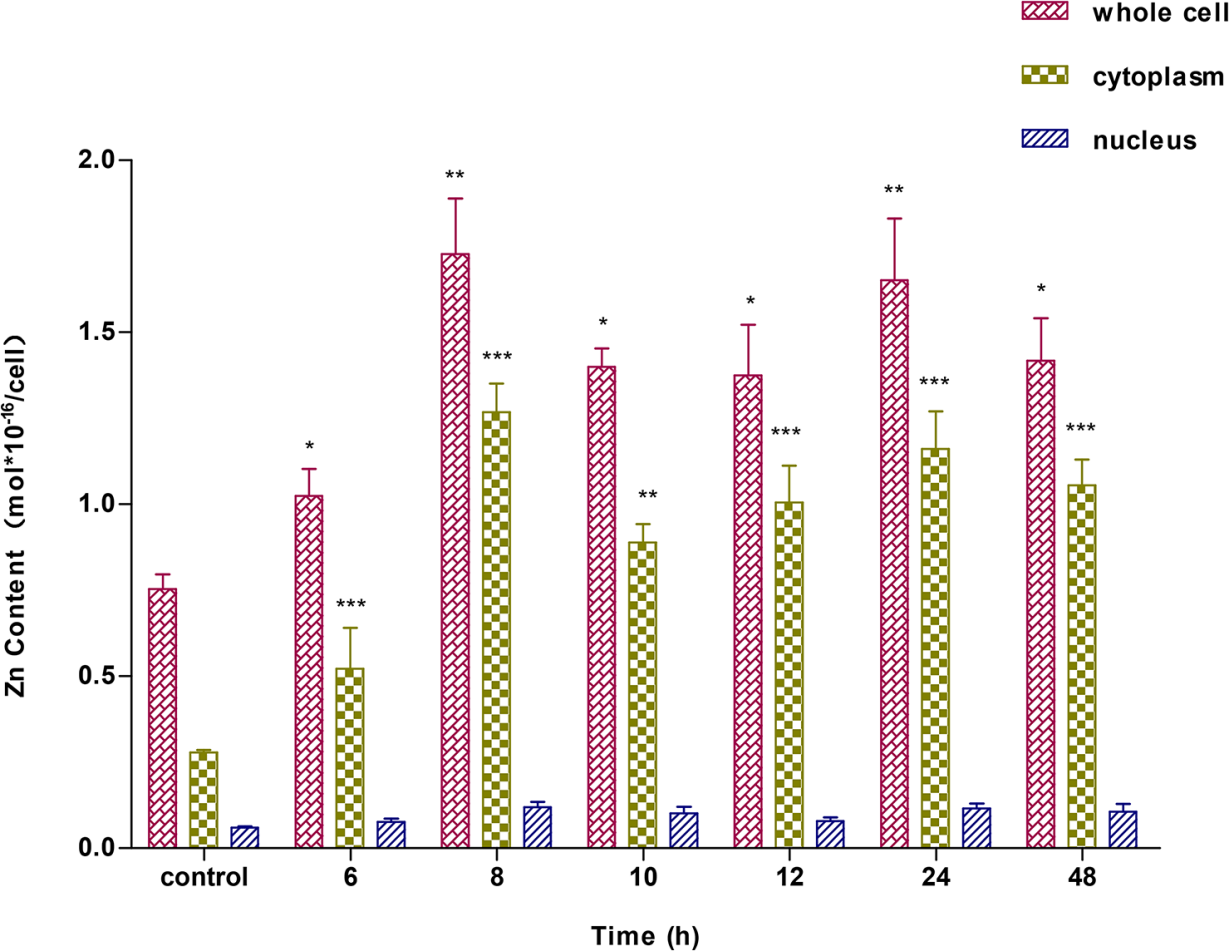
Changes in zinc content of A549 cells. A549 cells were incubated with 100 μM ZnSO_4_ for 0, 6, 8, 10, 12, 24, or 48 h. Cells were fractionated into cytoplasmic and nuclear fractions. Kinetic changes in the zinc contents were measured in whole cell, cytoplasmic, and nuclear fractions.

Intracellular zinc accumulation in A549 cells was measured by ICP-MS after treatment with ZnSO_4_ for 6, 8, 10, 12, or 24 h (Fig 8). Zinc contents in whole cells, cytoplasm, and nuclei fluctuated with treatment for different lengths of time and showed a similar trend. Zinc concentrations peaked at 8 h, and then decreased. Subsequently, zinc concentrations began to rise slightly at 24 h again. After this time-point, zinc concentrations also continued to decline. We detected significant differences in zinc concentrations in whole cells and cytoplasm after 6 h of treatment, whereas there were no significant differences observed in nuclei. Relatively low contents and activities of other metals in cells and environments could interfere with the precise determination of nuclear zinc content.

### Intracellular free Zn^2+^

**Fig 9:**
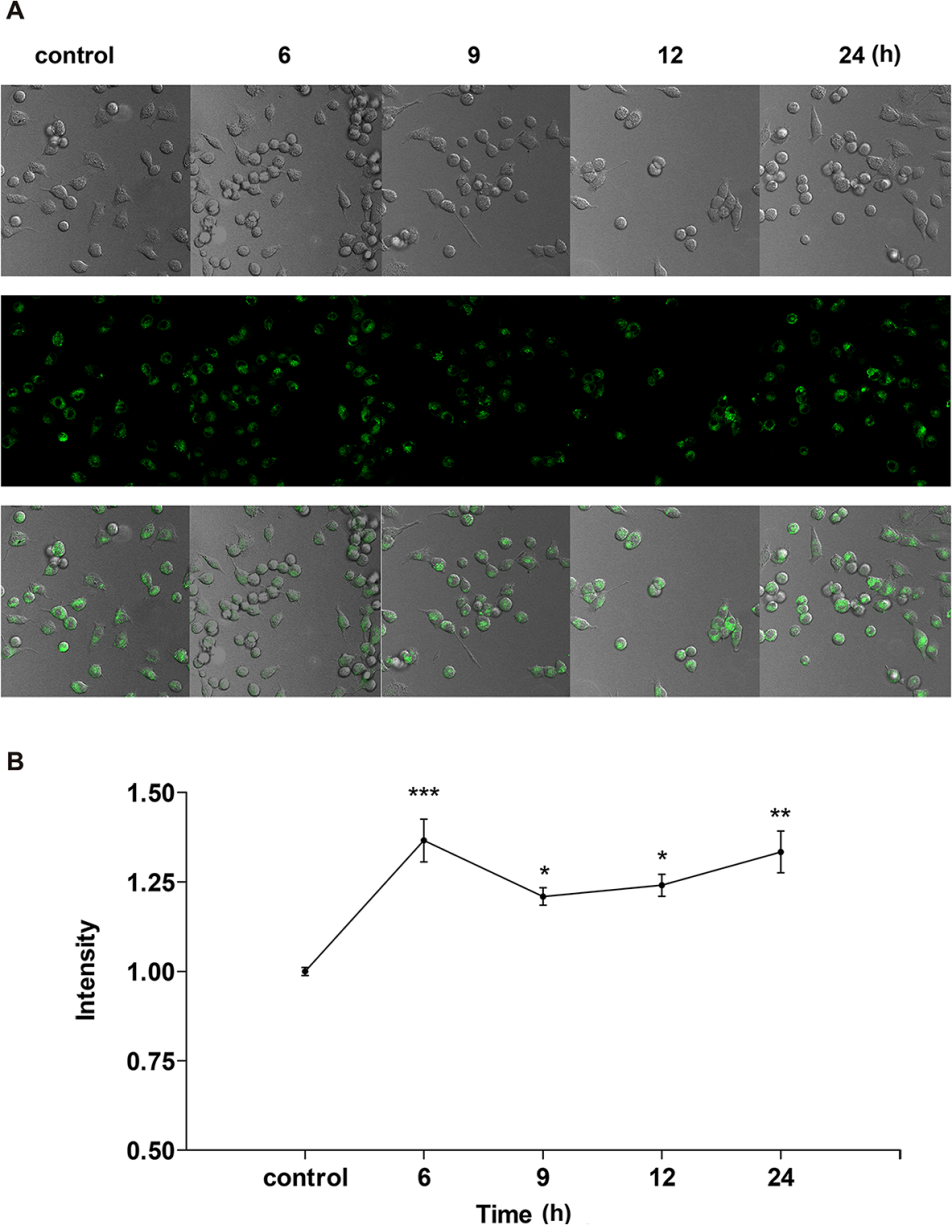
Confocal imaging of free Zn^2+^ in A549 cells after exogenous Zn^2+^ treatment for different periods. A549 cells were incubated with 100 μM ZnSO_4_ for 6, 9, 12, or 24 h, then were stained with 10 μM NBD-TPEA for 30 min. (A) Images were scanned with a 20× objective lens and were magnified by “zooming in” three-fold; (B) The fluorescence intensity of free Zn^2+^ was quantitatively analyzed by using Image-ProPlus 6.0 software. Data were normalized to controls.

Confocal microscopy was used to investigate the distribution and localization of free Zn^2+^ in A549 cells. Changes in intracellular Zn^2+^ after treatment with ZnSO_4_ for 6, 9, 12, or 24 h were observed using the highly selective and sensitive NBD-TPEA fluorescence probe. Confocal images and corresponding quantification analysis are shown in Fig 9. Owing to the necessity of free Zn^2+^ in normal cellular functions, cells that were not treated with exogenous zinc (controls) also showed fluorescence. Additionally, the fluorescence of Zn^2+^ in untreated cells showed an annular distribution. The green fluorescence intensity of Zn^2+^ became strong and spread around the annularity after treatment with ZnSO_4_. Furthermore, the fluorescence intensity was strongest after 6 h of exogenous zinc treatment among these time spans that we tested. After 9 h of zinc treatment, the fluorescence intensity around the annularity gradually decreased, and the annular spots appeared to gradually shrink into a few bright spots.

## Discussion

A549 cell responses to treatment with ZnSO_4_ occurred in a time-dependent manner and cell viability showed time-dependent changes. Sub-toxic ZnSO_4_ could trigger regulatory mechanisms of defense and/or homeostasis that guard against zinc stress. Additionally, we previously showed that the expression of zinc-regulated proteins exhibited a maximum change in protein and mRNA levels between 8 to 10 h, while slight changes could be observed at 24 h [35]. Therefore, 9 and 24 h time points of Zn^2+^ treatment were selected to compare differential expression in a time-dependent manner of zinc-responsive proteome in A549 cells and elucidate the zinc-responsive mechanisms and signaling pathways in A549 cells.

The 49 proteins spots showed robust reproducibility in differential expression with time-dependent expression patterns after Zn^2+^ treatment for both 9 and 24 h. The abundance of each protein spots varied in one of four patterns after Zn^2+^ treatment for different lengths of time. Notably, pattern 1 (up-regulated with rapid initial induction and subsequent repression) and pattern 4 (down-regulated with steady repression) were found to be the most prevalent change patterns. Among the proteins showing patterns 1 and 4, down-regulated proteins exhibited maximum changes at 24 h, while the up-regulated proteins exhibited maximum changes at 9 h of Zn^2+^ treatment. These trends indicate that an exposure time longer than 9 h would decrease the abundance of most differentially expressed proteins, irrespective of up- and down-regulation after Zn^2+^ treatment. Our previous result that the expression of proteins were regulated by MTF-1 exhibited a peak between 8–10 h during 24 h of Zn treatment corroborated this finding [35]. Therefore, small abundance ratios or the low expression of proteins in patterns 1 and 4 could be obtained compared with controls after 24 h of Zn^2+^ treatment. This finding indicates that a long exposure time of cells to exogenous zinc would impede the discovery of some important differentially expressed proteins and the elucidation of the molecular mechanism of zinc homeostasis and toxicity. Almost all differentially expressed proteins on 2DE gels stained by CBB displayed negative regulation after 24 h of Zn^2+^ treatment, which was found in our previous work and also supports this present conclusion [35]. CBB staining, although more suitable for quantification of 2DE gels because of its high reproducibility, is significantly less sensitive than silver staining, and only large changes in expression and highly abundant proteins are detected [25]. Therefore, both a higher LOD of CBB staining and a longer exposure time contribute to our previous finding that 17 of 18 differentially expressed proteins identified in our previous study showed marked suppression after Zn^2+^ treatment for 24 h, as indicated by the disappearance of these proteins from the developed gels [35]. Although differentially expressed proteins resulting from zinc exposure for 24 h or longer are more clinically significant, the proteins displaying large differences in expression from that early phase responding to zinc treatment should not be ignored. They maybe play critical roles in zinc homeostasis and toxicity. Additionally, more protein spots displayed differential expression and were identified successfully after 9 h of Zn^2+^ treatment than after 24 h, which also suggested that this duration of Zn^2+^ treatment was more advantageous for the observation and analysis of the differentially expressed proteome. Although determining the functional significance of these proteins will require further investigation, they undoubtedly play specific roles in zinc homeostasis.

Moreover, during our experimental time spans, proteins always showed relatively small changes of either up- or down-regulation. These conservative changes just with different ratios coupled with the four expression patterns (especially patterns 1 and 4), could facilitate the prediction of the time-dependent differential expression of uncharacterized proteins that respond to Zn treatment. For example, proteins Hsp90α and hnRNPA1 involved in cell physiology including zinc homeostasis by previous reports were found to be significantly up- and down regulated after Zn-treatment for 24 h, respectively (S1 File) [35, 43–46]. The changes in the expression of these two proteins after Zn^2+^ treatment over time conformed to pattern 1 (up-regulated with rapid initial induction and subsequent repression) and pattern 4 (down-regulated with steady repression), respectively. In addition, previous studies indicated that the expression of zinc-regulated key proteins, including MTF-1, MT-1, and ZnT-1, could be induced by Zn^2+^ treatment, and peaked after 8–10 h and then decreased [35], [47–49]. These key zinc-regulated proteins showed similar change to pattern 1, while the expression pattern of ZIP-1 involved in complex signaling pathways was consistent with pattern 4 [50]. Therefore, we make a bold proposal that the proteins grouped into patterns 1 and 4 were affected more directly by Zn^2+^ treatment and involved in less complex signaling pathways than the other differentially expressed proteins in pattern 2 (up-regulated with steady induction) and pattern 3 (down-regulated with rapid initial repression and subsequent induction). Accordingly, we not only could forecast the kinetic expression patterns of the two proteins, but also could speculate that they directly respond to Zn^2+^ treatment.

Additionally, we found that most differentially expressed proteins were induced by Zn-treatment, and moreover, that the magnitudes of up-regulation were greater than those of down-regulated proteins. Up-regulated proteins accounted for a large proportion of the 49 proteins and were almost three-fold greater than down-regulated proteins. Based on these findings, the concentrations of proteins after Zn^2+^-treatment for different lengths of time were always higher than controls. Furthermore, because of the same reduced expression tendency of most of proteins, including those up- and down-regulated after 9 h of Zn^2+^ treatment, the concentrations of proteins showed peak values between 8 to 10 h.

Characteristic cellular zinc ion concentrations support normal cell functions and avoid adverse effects. Zinc, a trace essential element, participates in many important molecular functions and biological processes [51]. Fluctuations or intracellular translocation of free zinc ions can regulate these molecular functions and biological processes [52]. Differentially expressed proteins identified by MS/MS and functional classification showed that the response of A549 cells to exogenous zinc stress were primarily involved in biological processes including metabolic processes, cellular processes, developmental processes and cellular component organization or biogenesis, and molecular functions including catalytic activity and binding and structural molecule activity. In fact, the regulation of bound zinc and free Zn^2+^ ions in cells is a collaborative and dynamic process [53]. When exposed to exogenous zinc ions, cells begin to rapidly initiate a buffering system and to maintain zinc homeostasis in cells. Exogenous Zn^2+^ treatment increases zinc ion concentrations in cells as a signal for MTF-1 to express zinc-responsive genes, such as MT-1 and ZnT-1, which counteract metal stress [47], [53]. The fluctuations of zinc ion level in cells must be controlled by the zinc transporters ZnTs (SLC30A) and ZIPs (SLC39A), which coordinate the availability and translocations of zinc ions [54]. Muffling describes the time-dependent changes that contribute to “buffering” in cells [55]. Time-dependent coordinated changes in expression of ZnT-1, ZIP-1, MT-1, and MTF-1 to maintain zinc homeostasis in A549 cells have been studied in our previous work [35]. Fluctuations of free zinc ions, total zinc, and protein expression in the cells is illustrated in Figs 7–9. Their peak values occurred after 6, 8, and 10 h of Zn^2+^ treatment, respectively. When exposed to exogenous Zn^2+^, zinc ion concentrations in the cells increased drastically, with the strongest fluorescence intensity of free zinc ions occurring after 6 h. Because the response to Zn^2+^ stress is not instantaneous, cells might accumulate zinc in excess of its levels of demand before the zinc-responsive changes in transporter activity could occur [11]. It was clear that the total zinc content reached a peak value after 8 h when zinc-responsive proteins had started to bind and expel excess Zn^2+^ to reduce the metal stress. Subsequently, zinc ion fluctuations gradually diminished after 9 h owing to strong buffering capacity and equilibration at new steady state within cells. The stimulation and binding of excess Zn^2+^ to zinc-regulated proteins in cells can activate and initiate the excess expression of zinc-responsive genes, resulting in the highest protein concentration after 10 h of Zn^2+^ treatment. At 8–24 h, zinc content in cells also exhibited relative low levels. Interestingly, the emerging fluorescent spots and secondary peaks observed in measuring total zinc after 24 h implies that the buffering capacity of cells, particularly the binding capacity to Zn^2+^ of zinc storing proteins in cells, such as MT-1, was gradually exhausted by continuous exogenous Zn^2+^ treatment. In response, cells had to transport excess zinc ions into the zincosome and organelles. Meanwhile, the proteome of cells adopted a passive response to zinc stress by reducing the expression of proteins. Cellular zinc ion fluctuations suggest that such modulations are physiologically significant.

## Conclusions

In this study, we profiled the proteome of A549 cells in response to exogenous zinc ion stress after different periods of time. Differentially expressed proteins after zinc treatment are predominantly involved in metabolic processes, cellular processes, and developmental processes. Dynamic changes in protein abundance of the differentially expressed proteome in cells revealed that the response of A549 cells to zinc stress has a noticeable time-dependent specificity that was distinct from the very well-known tissue and concentration-dependent effects. Moreover, the long duration of Zn^2+^ treatment would reduce the expression level of most proteins in cells, so small abundance ratios and/or low expression levels would mask the identification of some important differentially expressed proteins, which would impede investigations of the molecular mechanisms of zinc homeostasis and toxicity. Therefore, after selecting an optimal length of time for metal treatment, cells might show significant changes that originate from time-dependent adaptive responses to exogenous zinc ion exposure. Additionally, the grouping of four time-dependent expression patterns could help to predict and rationalize the kinetic differential expression of unfamiliar proteins in response to Zn^2+^ treatment. Therefore, when probing the responsive and molecular mechanisms of metal stress, paying more attention to time-dependent dynamic process should yield more robust data sets and enhance the efficiency of high throughput analysis by “omics” and system biological tools. Furthermore, time-dependent differential regulation of free zinc ions, total zinc, protein concentrations, and proteome expression in cells is a coordinated and dynamic process. Fluctuations or intracellular translocations of free zinc ions might control these complex zinc-responsive biological processes. The mechanism of “muffling” might explain these time-dependent changes. Consequently, our work provides a new perspective and a promising model for studying the metal-responsive proteome and elucidating the relevant molecular mechanism(s).

## Supporting Information

S1 Fig Classification for differentially expressed proteins after Zn^2+^ treatment for both 9 and 24 h. (TIF)

S1 File Differentially expressed proteins identified by MALDI TOF/TOF. Light cyan and light yellow shadows indicate proteins up- and down-regulated after Zn^2+^ treatment, respectively. The proteins which abundance ratio compared with controls at 9 h was higher than that at 24 h (i.e., Ratio_9h_ > Ratio_24h_) are in red font, and lower than that at 24 h (i.e., Ratio_9h_ < Ratio_24h_) are in black font. More protein spots with bigger abundance ratios at 9 h than that at 24 h were identified successfully, which also suggested that this duration of Zn^2+^ treatment was more advantageous for the observation and analysis of the differentially expressed proteome.
(XLS)

S1 Table Classification for alternative expression of other differential proteins. Total 25 protein spots showed significantly different responses (*p*<0.05, fold change>2) to Zn^2+^ exposure at both 9 or 24 h. This means that these proteins were detected with significant change compared with controls (*p*>0.05) or with the expression change of less than two-fold (fold change<2) after treatment for at least one group of 9 and 24 h. Among them, *p*-values for significant level of 4 proteins were less than 0.05 (*p*<0.05) for both 9 and 24 h groups, but the expression changes were less than two-fold (fold change<2) for either of these two groups. The numbers of these proteins are listed in the last two columns, among which, 3 protein spots exhibited lower expression after 24 h of treatment compared to 9 h, also indicating that longer stimulation mainly reduced the expression of differentially expressed proteins. The ratios of protein abundance were obtained by comparing the mean abundance in triplicate gels of corresponding differentially expressed proteins after treatment for 9 or 24 h with their controls using gel analysis software. Ratio values were higher than 2 for up-regulated proteins and ratio values were lower than 0.5 for down-regulated proteins.
(DOC)
