# Supplementary figures and images for: The Kinetic Response of the Proteome in A549 Cells Exposed to ZnSO4 Stress
Source: PLoS One. 2015 Jul 21;10(7):e0133451. doi: 10.1371/journal.pone.0133451 (PMC4510299; doi:10.1371/journal.pone.0133451)

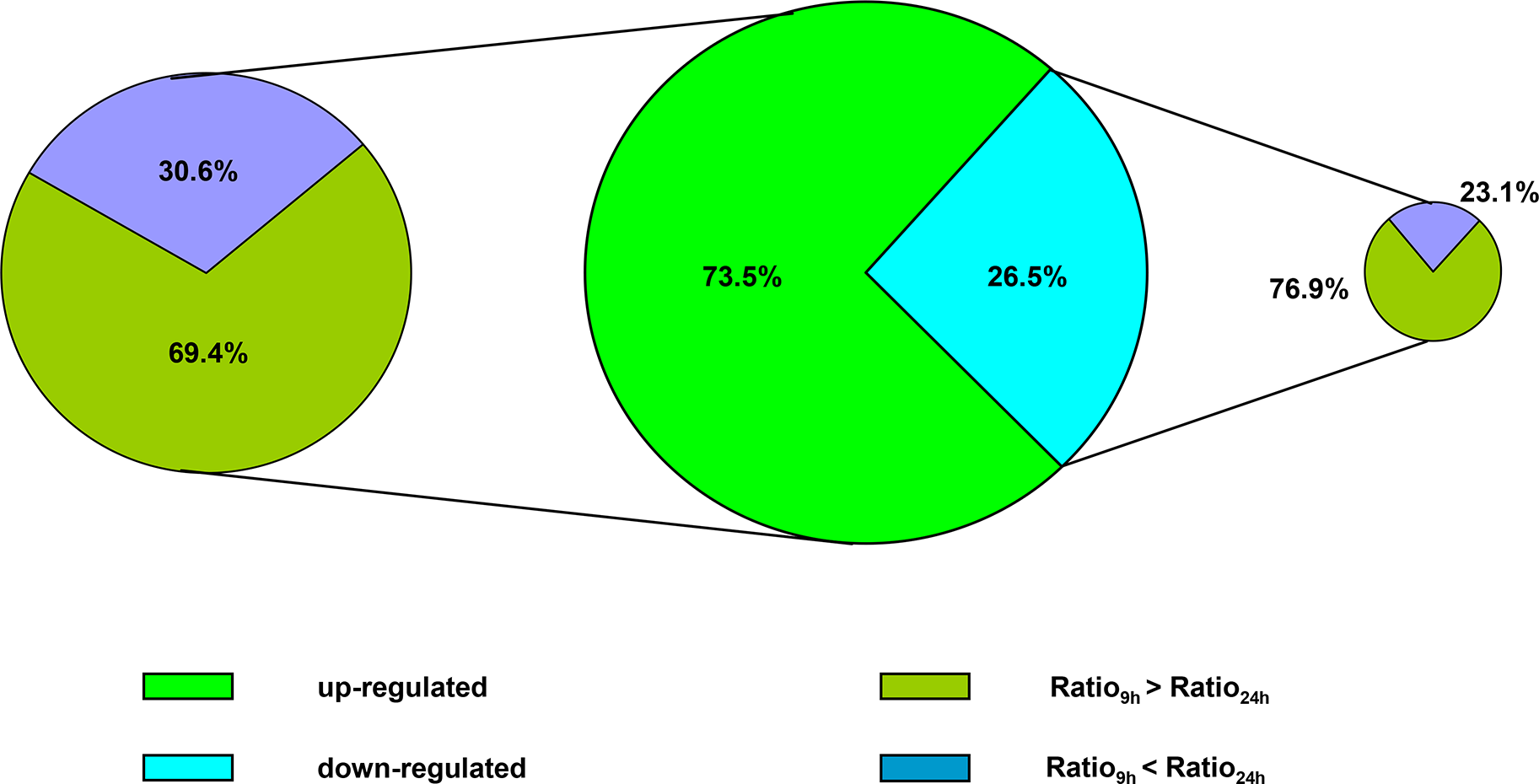

Supplement: S1 Fig — (TIF) [file pone.0133451.s001.tif]
