# Supplementary material for: The Kinetic Response of the Proteome in A549 Cells Exposed to ZnSO4 Stress
Source: PLoS One. 2015 Jul 21;10(7):e0133451. doi: 10.1371/journal.pone.0133451 (PMC4510299; doi:10.1371/journal.pone.0133451)
Supplement: S1 Table — Total 25 protein spots showed significantly different responses (p<0.05, fold change>2) to Zn2+ exposure at both 9 or 24 h. This means that these proteins were detected with significant change compared with controls (p>0.05) or with the expression change of less than two-fold (fold change<2) after treatment for at least one group of 9 and 24 h. Among them, p-values for significant level of 4 proteins were less than 0.05 (p<0.05) for both 9 and 24 h groups, but the expression changes were less than two-fold (fold change<2) for either of these two groups. The numbers of these proteins are listed in the last two columns, among which, 3 protein spots exhibited lower expression after 24 h of treatment compared to 9 h, also indicating that longer stimulation mainly reduced the expression of differentially expressed proteins. The ratios of protein abundance were obtained by comparing the mean abundance in triplicate gels of corresponding differentially expressed proteins after treatment for 9 or 24 h with their controls using gel analysis software. Ratio values were higher than 2 for up-regulated proteins and ratio values were lower than 0.5 for down-regulated proteins. (DOC) [file pone.0133451.s003.doc]

**Table S1. Classification for alternative expression of other differential proteins**

| Alternation | 9 h | 24 h | Total | Ratio_9h_ > Ratio_24h_ | Ratio_9h_ < Ratio_24h_ |
| --- | --- | --- | --- | --- | --- |
|  |  |  | (number) |  |  |
| Up-regulation | 11 | 2 | 13 | 2 | 0 |
| Down-regulation | 8 | 4 | 12 | 1 | 1 |
| Total | 19 | 6 | 25 | 3 | 1 |
